# Environmental control of social goals: using Pavlovian-to-instrumental transfer to test cue-based pro-self and pro-social outcome responses

**DOI:** 10.1098/rsos.220660

**Published:** 2023-01-25

**Authors:** Kaiyang Qin, Hans Marien, Ruud Custers, Henk Aarts

**Affiliations:** Department of Psychology, Utrecht University, Utrecht 3584 CS, The Netherlands

**Keywords:** goals, monetary rewards, self-interest, other-interest, Pavlovian-to-instrumental transfer

## Abstract

A large amount of literature demonstrates that social behaviour can be triggered by environmental cues. A long-standing debate involves the question of whether such stimuli trigger behaviour directly (i.e. habits) or whether these effects mediate goals. As studies on automatic goal pursuit typically use real-world cues that are already associated with the behaviour and potentially the goal, it is impossible to make strong claims about the nature of the effects. In the present paper, we use a paradigm inspired by the Pavlovian-to-instrumental transfer (PIT) literature to examine how the environment can trigger goal-directed behaviour. Building on the essence of pro-self and pro-social motives in humans, two experiments explored the PIT effect when the outcomes were framed in terms of self- versus other-interest. Participants performed actions to earn money for themselves or a charity. Each outcome was linked to a different cue. The results showed that a cue predictive of self-interest outcomes facilitated responses instrumental in gaining the outcome, while such specific PIT effect for other-interest outcomes only emerged when participants were free to donate the money. We briefly discuss these findings reflecting on whether the PIT effect in our paradigm is indeed sensitive to the value of social goals.

## 1. Introduction

An important question addressed in psychology is how the environment can control behaviour. The notion that behaviour is shaped by environmental stimuli is key to behaviourist approaches to habit learning [1,2], according to which habits are formed when a motor behaviour (e.g. pressing a lever) is repeatedly executed in response to a stimulus and reinforced by a rewarding outcome (e.g. a drop of sweet water). Whereas habit accounts have dominated the understanding of environmental control of behaviour for many years [3], defining habits as stimulus-response (S-R) links, more recent studies reveal that stimuli can trigger behaviour indirectly. According to this research on automatic goal pursuit, stimuli can activate mental representations of rewarding outcomes that facilitate the execution of actions instrumental in attaining those outcomes [4–7]. In other words, stimuli in the environment can act as cues for rewarding outcomes. Environmental control of behaviour, then, seems to be mediated by representations of rewarding outcomes and can therefore be regarded as goal-directed.

Although in the classic work on environmental control of behaviour, rewarding outcomes are usually related to eating and drinking [8–11], people often pursue rewarding outcomes that are social in nature [12,13]. In general, the social goals that people aim to attain are directed toward two types of outcomes: goals that serve self-interest (pro-self outcomes) and goals that serve the interest of others (pro-social outcomes). For instance, people might spend their money for their own benefits (e.g. buying valuable consumer goods to increase their social status) or share it with others (e.g. donating to a charity to help people in need). It is not clear, however, whether such social goals and resulting behaviour can be controlled by the environment as well.

Understanding cue-based control over people's engagement in pro-self or pro-social behaviours is highly relevant for various societal issues, such as social inequality, public health hazards and environmental issues. It has been suggested that especially when goals are repeatedly and consistently pursued by instrumental actions, activating the representation of the goal may trigger these instrumental actions [14,15]. This way, people would engage in goal-directed behaviour (e.g. spending money on oneself or others) without much deliberation and thought. Recent review studies reveal that behaviours linked to social goals can indeed be activated by the environment, and these effects are stronger for actions that produce more valuable outcomes [16,17]. Therefore, cue-based control of behaviour may not be understood as purely habitual but as mediated by activating representations of desirable outcomes or goals.

It has been pointed out, though, that while these studies hint at the involvement of goal representations, previous work on automatic goal pursuit does not properly distinguish between behaviour that is activated directly by the cues and behaviour that is mediated by representations of desired outcomes [3,4,18]. That is, the cues used in these studies are usually associated with both the outcome and the instrumental actions leading to them in daily life. Therefore, it is impossible to distinguish between direct and indirect effects of cues on behaviour. To address this shortcoming, we turn to the literature on Pavlovian-to-instrumental transfer (hereafter abbreviated as PIT), which addresses this question in animal research [8–11] precisely, but also in humans, albeit with non-social behavioural outcomes [19–21].

Here, we report an initial test inspired by the classic PIT paradigm that examines whether environmental cues can trigger behaviour through pro-self and pro-social goals. Fundamentally speaking, cue-based goal-directed behaviour relies on two distinct learning processes. First, people need to represent their behaviour in terms of outcomes so they can anticipate these outcomes and direct their actions accordingly [22]. Second, people need to understand that the cue represents an opportunity to obtain the outcome in the current situation [23,24]. The classic PIT paradigm experimentally separates these processes into two learning phases: the instrumental learning phase, in which the response is learned to produce an outcome, and the Pavlovian learning phase, in which a cue is learned to predict the same outcome. This way, the Pavlovian cue and response share the same outcome, allowing people to mentally link the cue and response through the shared outcome representation. The typical finding is that subsequent exposure to the cue facilitates the instrumental response (e.g. perform the response faster or more frequently), even though the response is never executed in the presence of the cue. The explanation for this effect is that the cue activates the outcome representation, which in turn triggers the instrumental response: the PIT effect. Importantly, using two distinct test procedures (i.e. outcome devaluation and high- versus low-value outcome comparison), PIT seems more pronounced for outcomes that are represented as having high value, indicating that relatively strong (versus weak) desirable goals are more likely to be controlled by the environment [25]. These value-based PIT effects have been demonstrated initially in animals (e.g. [9,10]) but lately also in humans (e.g. [19–21]).

Pro-self and pro-social motives are central to everyday human interactions, particularly people's goal to earn money for themselves (self-interest outcomes) versus earning money for others (other-interest outcomes). A well-established observation is that people are highly motivated to seek rewards for self-interest purposes [26–28]. The rationale underlying this notion relies on classical conceptions of self-interest from evolutionary biology (genetic selfishness to increase an organism's fitness), economics (i.e. rational self-interest) and philosophy (i.e. psychological egoism). The general gist here is that pro-self goals are dominant in social contexts that yield a conceivable self-benefit, such as earning money for oneself. By contrast, it seems less important for individuals to earn money for others, especially when financial rewards for themselves (self-interest outcomes) are available [29,30]. Hence, when individuals can attain self-interest and other-interest goals within the same context, they are likely to attach a higher value to self-interest outcomes than other-interest outcomes.

Evidence that the environment can trigger self-interest-oriented behaviour comes from a recent meta-analysis on the concept of automatic behaviour [16]. Analysing data from 133 studies, this study suggests that behaviour that serves self-interest can be triggered automatically by cues that refer to pro-self goal concepts such as striving for power, status or personal achievement. Such cue-based behaviour is more pronounced when the goal is perceived as a valuable outcome. Furthermore, whereas pro-self behaviour might be the default in the social context that clearly yields a self-benefit outcome, there is some research showing that cuing concepts related to pro-social goals (e.g. cooperation, helping) influences the decision made in these settings in favour of the pro-social outcome, as revealed by increased helping or donation behaviour (e.g. [31–34]). Such pro-social automatic goal-pursuit might stem from intuitive processes shaped by successful strategies in social interactions and the internalization of cultural norms [35].

It is important to note that previous studies on pro-social automatic goal-pursuit suffer from a few methodological weaknesses that undermine the conclusion that cue-based social behaviour is mediated by pro-self or pro-social goals. In a typical experiment, participants are exposed to words (e.g. power, help) or pictures (e.g. a $10 note) that are assumed to trigger goals associated with them. Effects of the word or picture exposure (compared with a control condition) are then tested on the speed or direction of goal-related behaviour using a response time or choice task. Furthermore, in some studies, the value of the goal is measured as an individual difference variable to examine whether automatic goal pursuit is more pronounced for goals that have value and matter to people. The automatic goal-pursuit effects are interpreted as being caused by the mediation of the mental representation of the goal. The words or pictures are supposed to trigger the representation of the high-valued (versus low-valued) outcome, which in turn activates the resulting action. Although suggestive, these results are not conclusive regarding the mediating role of goals: the participants in these studies show performance on a behavioural measure after seeing words or pictures, but these effects do not attest to the basic assumptions that are argued to underlie cue-based goal-directed behaviour. That is, we do not know for sure whether participants did learn (i) to represent their behaviour in terms of high-valued outcomes that they produced by the behaviour and (ii) that the cue predicts the same outcome. We argue that previous research did not take these basic learning aspects sufficiently into account and suggest that a PIT approach may provide an important additional test to examine whether cue-based behaviour is mediated by the representation of pro-self or pro-social goals.

Most PIT studies with human subjects rely on a decision-making task, asking participants to select one of two actions in response to a Pavlovian cue. In this context of choice options, decisions usually depend on preferences and framing in the presence of decision-relevant cues (e.g. [36,37]). Choice measures thus represent strategic behaviour resulting from explicit beliefs and expectations that can bias choices in the PIT task due to demand characteristics [38]. To rule out such strategic decision process, a few studies have tested value-based PIT effects in a cue-based response time task [38,39]. For example, Qin *et al*. [38] designed a cue-based forced-choice response time PIT test that measures the speed of responding with instrumental action in the presence of Pavlovian cues. This measure thus does not rely on a decision-making process but capitalizes on response facilitation upon being exposed to response-related cues. Employing a high- versus low-value outcome comparison procedure, participants could press a left or right key to earn low- or high-value monetary rewards. The rewards (coins of 5 Euro cents or 20 Euro cents) were presented on the computer screen each time they pressed the correct key. In a Pavlovian learning task, participants learned to associate the two different coins with two new neutral cues. In the following test task, participants were briefly exposed to one of the Pavlovian cues, followed by the requirement to press the left or right key as fast as possible. Results of the test task showed that participants responded faster when the response and the cue predicted the same outcome, while the response was not directly executed in response to the cue. Moreover, this effect was more pronounced for high-value (versus low-value) outcomes, thus showing a value-sensitive specific PIT effect. Similar effects have been found with multiple food outcomes that differ in value (e.g. Watson *et al.* [39]).

We report two experiments that adapted the procedure of Qin *et al*. [38] to the context of pro-self and pro-social goals. In both experiments, using a within-subject design, participants could earn a small monetary reward that had either self-interest or other-interest meaning. The self- versus other-interest meaning was manipulated in instrumental and Pavlovian learning phases. Specifically, in Experiment 1, participants pressed the left (R1) and right (R2) keys, which resulted in earning 10 Euro cents coin for themselves (O1) or for others by donating it to a charity (O2). Furthermore, in the Pavlovian learning phase, each outcome was linked to one of two stimuli (S1 and S2). Accordingly, the Pavlovian cue (S1) and response (R1) share the same outcome (O1), and the Pavlovian cue (S2) and response (R2) share the same outcome (O2). Importantly, participants do not learn to respond with R1 to S1 or with R2 to S2. They can, however, mentally link the cue and response through the shared outcome representation. This allowed us to test whether a Pavlovian cue associated with self-interest versus other-interest outcome will speed up the corresponding response in the forced-two response time test [38]. If the cue associated with the self-interest outcome speeds up the respective instrumental response, but the cue associated with the other-interest outcome does not, this will demonstrate that cues mainly trigger pro-self (versus pro-social) goals and resulting behaviour as they are represented as high-value self-interest outcomes, at least in the value-based PIT framework. Experiment 2 aimed to explore whether we can render other-interest outcomes as valuable as self-interest outcomes, providing further evidence that the social meaningfulness of the value of the same monetary reward can produce specific PIT effects.

## 2. Experiment 1

In Experiment 1, we examined whether participants' responses are facilitated by Pavlovian cues predicting a single valuable outcome and whether this effect is stronger for self-interest represented outcomes than other-interest represented outcomes. The self-interest outcome was operationalized as earning 10 Euro cents for oneself, whereas the other-interest outcome was operationalized as earning 10 Euro cents for a charity, namely the Against Malaria Foundation (a foundation that can provide mosquito nets from the donations). We chose this charity because donations are typically provided in small amounts of money (i.e. €2), which enabled participants to earn enough money during the experiment to provide an effective donation. Given that the self-interest and other-interest outcomes both consist of the same objective reward before the test phase (i.e. both are 10 Euro cents coins), but the self-interest outcome is considered to have more subjective value than the other-interest outcome in the current context, we expected a cue-based facilitation effect to occur for the self-interest outcome but not for the other-interest outcome.

### 2.1. Method

#### 2.1.1. Participants and design

Participants were recruited by posting advertisements that targeted English-speaking students under the age of 35. The required sample size for testing the cue-based facilitation effect was determined using G*Power analysis [40]. We aimed to detect a medium effect size ($\eta_{p}^{2}=0.10$; based on previous research, Qin *et al*. [38]) with a power of 0.80 and used three measurements for the 2 × 3 within-subjects design test and epsilon = 1. The sample size analysis indicated that in the current experiment, at least 46 participants were needed. Concerning the possible dropout, we decided to recruit two more participants. Finally, 48 participants (mean age 24.5; 34 females) were recruited. We excluded data from one participant for not following the instructions. The remaining participants participated in the experiment with a 2 (Response outcome: self-interest versus other-interest) × 3 (cue outcome: neutral versus self-interest versus other-interest) repeated measures design. The neutral cue was used as a baseline to control for differences between the speed of self-interest and other-interest responses. Participants received a show-up fee of €2 and could earn €4 (€2 extra for themselves and €2 extra for the charity).

#### 2.1.2. Apparatus and materials

Participants were placed at a desk in a soundproof cubicle facing a computer screen, and a standard keyboard was in front of them. The experiment was programmed in Matlab with Psychophysics Toolbox v. 3.0.10 [41]. The monitor screen (1920*1080 pixels) presented a black background and projected instructions in white. During the task, a grey square (RGB 192 192 192, visual angle 6.60°) appeared in the centre of the screen. Three simple figures (i.e. a ‘star’, a ‘cloud’ and a ‘moon’, visual angles 6.60°) were presented in the centre of the grey square. A yellow frame (RGB 255,255,0 visual angle 6.86°) and a blue (RGB 0,0,255 visual angle 6.86°) frame surrounding the grey square appeared as prompts for responses. The self-interest and other-interest outcomes used in the learning phases were depicted by a full-colour image of a 10 Euro cents coin dropping in a piggy bank. To support participants in representing the money as being self- versus other-relevant the word ‘ME’ (representing the self-interest outcome) or ‘NETS’ (representing the other-interest outcome) was printed on it. The word NETS was used to refer to the mosquito nets that the Against Malaria Foundation can buy from the donations.

#### 2.1.3. Procedure

Participants signed the informed consent upon arrival at the laboratory. The experimenter told participants that the study contained several tasks, and they could earn extra money for themselves and the Against Malaria Foundation to help people under the threat of malaria (see electronic supplementary material for complete information about the charity provided to participants). The experimenter stayed in the cubicle during the entire experiment and sat behind a divider screen to monitor the procedure and task performance of the participant during the experiment. The experiment contained four phases: a demonstration phase, an instrumental learning phase, a Pavlovian learning phase and a test phase.

##### 2.1.3.1. Demonstration phase

The experiment started with a demonstration task to familiarize participants with the speeded response task (for details see the test phase). They performed 42 trials in total.

##### 2.1.3.2. Instrumental learning phase

Participants learned that they could earn money for themselves (the self-interest outcome) or a charity (the other-interest outcome) by correctly producing two different motor responses. Participants first practised 20 trials (block 1), followed by 20 real trials (block 2). The trials in the practice and the actual task were randomly presented, and each condition (i.e. the self-interest outcome response and the other-interest outcome response) was repeated 10 times in each block.

**Figure 1.**
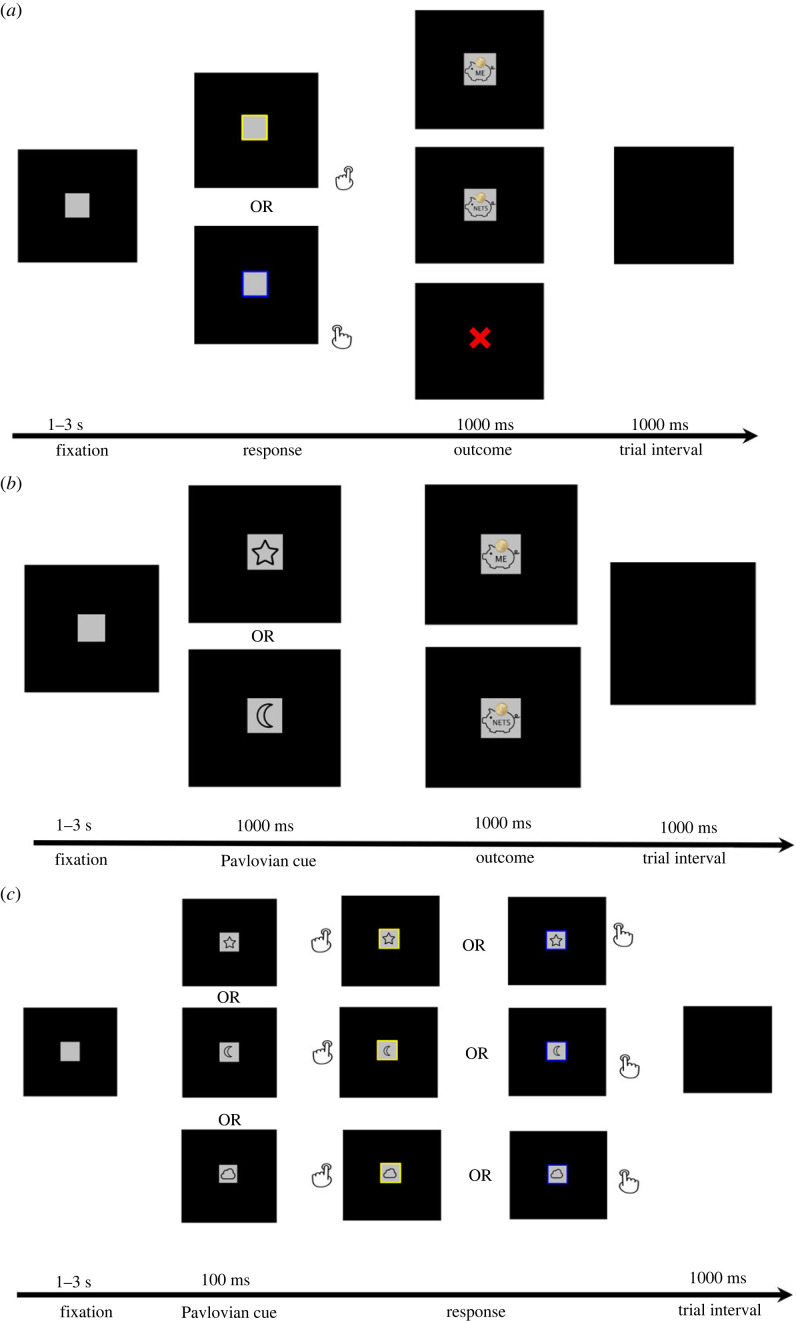
Flowchart of the correct response in instrumental learning (*a*), Pavlovian learning (*b*), test phase (*c*).

The trial procedure is depicted in figure 1 (panel *a*): Each trial started with a grey square for 1–3 s (random time interval), then a yellow or a blue frame indicated to press the left or right key. Participants could earn 10 cents for themselves by correctly pressing the (left) ‘s’ key (yellow frame) and 10 cents for the charity to buy malaria nets by correctly pressing the (right) ‘k’ key (blue frame); coloured frames were counterbalanced across participants. After a correct keypress, the self-interest outcome or other-interest outcome (represented by the picture of a 10 cents coin dropping in a piggy bank) was presented for 1 s. To support participants in keeping the self- versus other-interest outcome in mind, the picture of a 10 cents coin and a piggy bank also displayed the word ‘ME’ or ‘NETS’. To encourage participants to carefully process the outcome information, they had to speak out ‘10 for ME’ or ‘10 for NETS’ when pressing the corresponding keys. The experimenter took note of whether participants spoke out the correct outcome.

Participants did not know how many trials they had to do and how many they had executed correctly. After the task, they were told how much money they had earned. We decided to give all participants the same amount of extra money: €2 (€1 for themselves and €1 for the Against Malaria Foundation). Actual earnings thus were independent of performance.

##### 2.1.3.3. Pavlovian learning phase

In this phase, participants learned that they could earn money for themselves or the charity in a cue–outcome learning task. Participants performed 40 trials (two blocks); the first half was practice trials (block 1), and the second half was the actual trials (block 2). The practice and the actual trials were randomly presented, and each condition (i.e. the self-interest outcome cue and the other-interest outcome cue) was repeated 10 times in each block. Participants could only earn coins for themselves and the charity in the actual task.

The trial procedure was as follows (figure 1, panel *b*): A grey square appeared for 1–3 s (random time interval), then one of two cues (e.g. a ‘star’) appeared for 1 s. Like in the instrumental phase, participants could earn 10 cents for themselves when they saw a ‘star’ and 10 cents for charity when they saw a ‘moon’ (the particular self–other mapping was counterbalanced across participants). To encourage participants to process the outcome information carefully, they were asked to speak out ‘10 for ME’ or ‘10 for NETS’ when they saw the corresponding cues. The picture of the self-interest or other-interest outcomes (‘ME’ piggy bank or the ‘NETS’ piggy bank) was presented for 1 s. Note that while naming rewards in this task could be regarded as partly instrumental, this vocal behaviour was not instrumental in obtaining the reward (participants simply had to comply with the instructions). The experimenter took notes on whether they spoke out the correct outcome in response to the cues.

Like in the instrumental learning phase, participants did not know how many trials they had to do and how many they had executed correctly. After the task, they were told how much money they had earned. We again decided to give all participants the same amount of extra money: €2 (€1 for themselves and €1 for the Against Malaria Foundation). Actual earnings thus were independent of performance. Accordingly, in total, participants earned an additional €4 for performing the instrumental and Pavlovian task (i.e. €2 for the participant and €2 for the charity).

##### 2.1.3.4. Test phase

In this phase, participants were informed that they could not further earn money. They were asked to respond as quickly and accurately as possible with the left or right keypress in a series of response time trials. The trial procedure of the speeded response task was taken from Qin *et al.* [38] and looked as follows (figure 1, panel *c*): each trial started with a grey square, followed by one of the three cues (star or moon or cloud) which appearing inside the grey square after a 1–3 s (randomized time-interval). After 100 ms, a coloured frame appeared on the computer screen surrounding the grey square, thus prompting participants to press the left or right key (counterbalanced). The Pavlovian cue remained on the screen until a response was given.

In the test phase, the cues (star and moon) that were learned to be associated with self-interest versus other-interest outcomes (or vice versa) were combined with the responses (pressing s and k keys) that were also learned to be associated with self-interest versus other-interest outcomes. To iterate, then, a cue-based facilitation effect emerges when the self-interest cue speeds up the self-interest response, while such a speed-up effect is not expected for other-interest responses that are preceded by other-interest cues. A third neutral cue (e.g. a cloud) served as a baseline condition. This cue was not learned to be associated with any of the outcomes, thus allowing us to check for response time (RT) differences between self-interest and other-interest responses that are independent of Pavlovian cues. There were 120 trials (four blocks) in total. The trials were randomly presented, and each condition was repeated five times in each block.

After the experiment, participants were thanked and received €6 (€2 for showing up and €4 for their performance in the instrumental and Pavlovian learning phases). Participants received this amount in 50 cents, €1 and €2 coins and were told they could donate their money to the Against Malaria Foundation if they wished to do so. A donation box was placed in the experiment room, which had not been visible to the participant during the experiment. To prevent social pressure from the experimenter's presence, the experimenter left the room for 20–30 s, during which participants could decide to either keep €6 for themselves or donate some (or all) of it. In total, participants donated €61 to the Against Malaria Foundation, which accounted for 63.5% of the initially reserved amount for donation according to the earnings in the learning phases (i.e. 48 participants * €2 for charity = €96). We donated the €61 on behalf of the participants to the Against Malaria Foundation.

#### 2.1.4. Data preparation and analyses

We trimmed the RT data of the correct responses in the test phase for outliers [42] and removed data points that were 3 s.d. slower or faster than that of the participant's mean RTs (4.2% of the RTs). We did the trimming because it is typically applied for analyses of stimulus-response compatibility effects (e.g. Theeuwes *et al.* [43]), and here we followed the same trimming procedure as in the previous research [38]. Since the RT and accuracy data were not normally distributed, we performed a reciprocal transformation (i.e. 1/x) to normalize the distributions (for details of the normality test of the two experiments, see the electronic supplementary material), and we used the transformed data for further tests.^1^

Considering that the conventional 2*3 repeated measures ANOVA may not capture the predicted pattern for RT, we performed a planned contrast to the RT difference in three cue conditions using an *F*-test with partial eta squared ($\eta_{p}^{2}$) as effect size, which is reported with a 90% CI [44,45]. Typically, participants should respond more readily when the cue and the response predict the same valuable outcome. Accordingly, if representing the outcome (10 cents) from a self-interest (versus other-interest) point of view enhanced the subjective value of the outcome, then the cue-based facilitation effect should mainly occur in the self-interest outcome condition. This means that the RT difference between the self-interest and other-interest response should be larger in the self-interest cue condition compared with the neutral cue and the other-interest outcome cue condition. Because the other-interest representation is expected not to enhance the value of the outcome, the responses in the neutral and other-interest cue conditions will not differ.

To test this, we subjected the RT differences (self-interest minus other-interest responses) to a repeated ANOVA with neutral, self-interest and other-interest cues as a within-subject factor. Note that a negative RT difference score represents a facilitation effect for responses that lead to self-interest outcomes, and a positive one represents a facilitation effect for responses that lead to other-interest outcomes. We tested these effects according to the following contrast: +1 for the RT difference in the neutral cue condition, −2 for the RT difference in the self-interest outcome cue condition, and +1 for the RT difference in the other-interest outcome cue condition. We used the same approach as the analysis of RTs for the accuracy data analysis but reversed the contrast coding weight because participants should respond more accurately when the cue shares the identical outcome representation with the response. Note that a positive accuracy difference score represents more correct responses towards self-interest outcomes, and a negative one indicates more correct responses that lead to other-interest outcomes.

### 2.2. Results

#### 2.2.1. Instrumental learning phase

The results of the instrumental learning phase indicate that no difference was found on RTs^2^ (*t*_46_ = 0.76, *p* = 0.449), but the test on accuracy (*t*_46_ = 3.15, *p* = 0.003, Cohen's *d_z_* = 0.46 [0.158; 0.766]) indicates that participants responded more accurately on the other-interest response than the self-interest response in the instrumental training phase.

#### 2.2.2. Reaction times in the test phase

**Figure 2.**
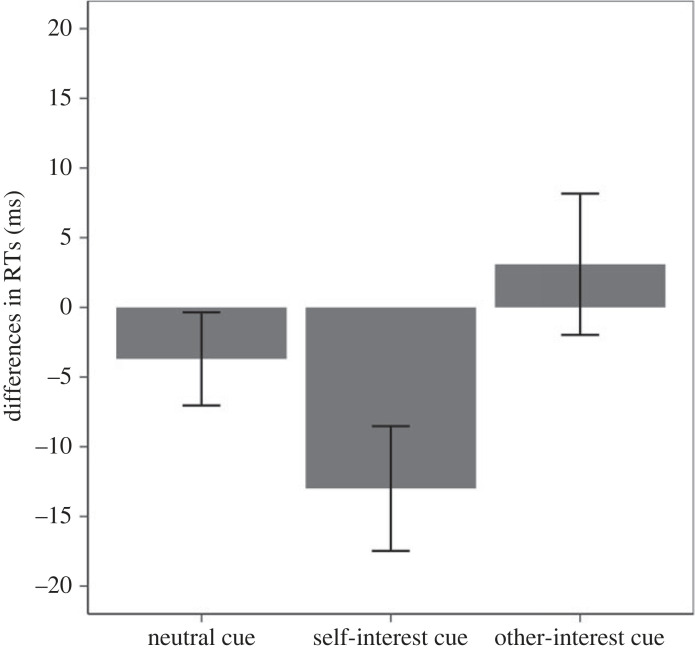
Experiment 1 RT difference in three cue conditions (error bars represent one standard error). Note: a negative score represents faster self-interest responses, and a positive score represents faster other-interest responses.

The pattern of reaction time difference is presented in figure 2. The planned contrast was significant (*F*_1,46_ = 5.82, *p* = 0.020, $\eta_{p}^{2}=0.11$ [0.010; 0.267]). In line with predictions, the RT difference score between the self-interest outcome and other-interest outcome responses is larger and negative in the self-interest cue condition compared with the other two conditions. Furthermore, the RT difference scores do not seem to differ between the neutral and other-interest cue conditions.

#### 2.2.3. Accuracy in the test phase

**Figure 3.**
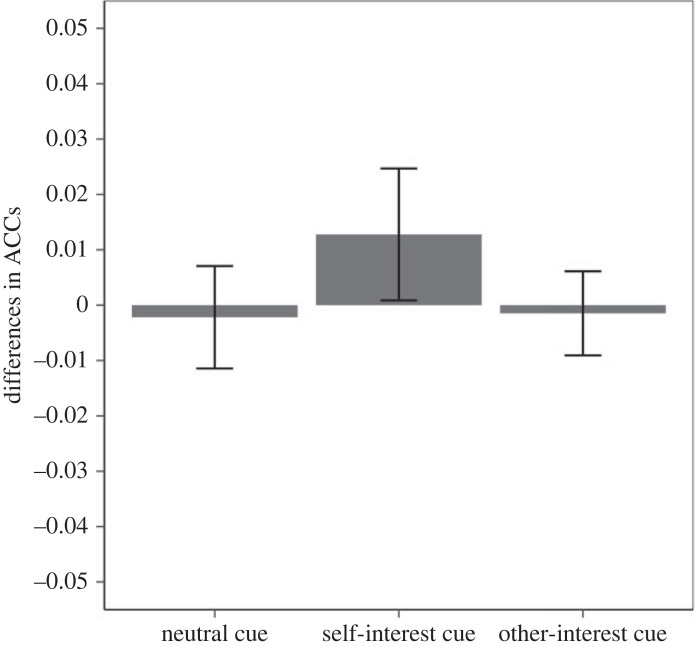
Experiment 1 accuracy difference in the three cue conditions of the test phase (error bars represent one standard error). Note: a positive score represents more accurate self-interest responses and a negative score more accurate other-interest responses.

The planned contrast did not yield the predicted pattern for accuracy (*F*_1,46_ = 1.04, *p* = 0.313). Figure 3 presents the means of the accuracy scores in each cell of the design. Please note that, if anything, the pattern of accuracy shows that participants responded more accurately to the self-interest response (versus other-interest response) when encountering the high-outcome-value cue. This suggests that the RTs effect cannot be easily explained by a speed–accuracy trade-off.

### 2.3. Discussion

Experiment 1 showed that the same monetary reward caused a cue-based facilitation effect when that reward was earned for oneself rather than others. Considering that an effect follows from a value-based account, according to which PIT is sensitive to the value of the outcome, our findings suggest that representing the collection of 10 Euro cents in terms of being instrumental for oneself enhances the subjective value and motivation to attain it. Circumstantial evidence for this comes from the donation amount at the end of the experiment, showing that participants only paid a fraction of what they had earned in the two learning tasks. Such behaviour is in line with the rich tradition of research showing that self-interest is a powerful motive for human behaviour associated with personal gains [28,46,47].

Our findings from Experiment 1 also show that a cue-based facilitation effect did not emerge when participants represented the behaviour of earning coins in terms of donating them to the Against Malaria Foundation. From a community point of view, it would be helpful if PIT is also sensitive to behaviour directed at other-interest outcomes. Research suggests that individuals can prioritize the interests of others [35,48,49]. However, it is yet unclear why the other-relevance framing did not produce a facilitation effect, as is revealed by the observation that the RTs for the neutral cue and other-interest outcome cue did not differ.

It is important to note that, in the current task, the other-interest outcome was specifically targeted at the Against Malaria Foundation, whereas the self-interest outcome could be used for any self-interested cause that participants had in mind. In other words, participants had no freedom of choice for utilizing the other-interest (versus self-interest) outcome. Choice freedom is essential to intentional action [50] and a strong internal motivator for behaviour [51]. No choice freedom may have caused participants to consider the other-interest outcome less valuable compared with the self-interest outcome, thus causing the cues related to other-interest to evoke less of a facilitation effect, which is in line with the outcome value-based PIT effect [38]. Accordingly, if choice freedom is central to cue-based facilitation effects of self-interest goals, then adding such freedom might also render the effect sensitive to the value of other-interest goals. We designed a second experiment to explore this further.

## 3. Experiment 2

In Experiment 2, we did not specify which charitable fund one could donate to but offered participants the opportunity to select one themselves. This way, the other-interest outcome would share the same characteristic of freely spending the earned money as the self-interest outcome in one single context, thus inducing a fairer comparison in terms of value between the two outcomes. Based on the reasoning about the choice of freedom addressed above, we expected that the cue-based facilitation effect should be observed for both self-interest and other-interest outcomes, such that self-interest cues and other-interest cues speed up self-interest responses and other-interest responses, respectively.

### 3.1. Method

#### 3.1.1. Participants and design

We increased the sample size to obtain a more sensitive measure for detecting a cue-based facilitation effect following the findings of Experiment 1. We recruited 60 participants (mean age 23.43 (s.d. = 3.76); 45 females). Data from two participants were excluded from the analysis: one participant indicated having already participated in Experiment 1, and one participant had excessively low accuracy (less than 3 s.d. from sample mean). The remaining 58 participants participated in the experiment with a 2 (Response outcome: self-interest versus other-interest) * 3 (cue outcome: neutral versus self-interest versus other-interest) repeated measures design. Participants received a show-up fee of €2 and could earn €2 extra for themselves and €2 extra for the charity.

#### 3.1.2. Apparatus and materials

The materials used in Experiment 2 are the same as in Experiment 1 except for the framing of the outcomes. The self-interest and other-interest outcomes that appeared in the learning phases were depicted by a full-colour image of a 10 Euro cents coin dropping in a piggy bank with either the word ‘ME’ (representing self-interest outcomes) or ‘FUND’ (representing other-interest outcomes) printed on it, respectively. The latter word was used to refer to the possibility of spending the money at any charitable fund one likes.

#### 3.1.3. Procedure

The procedure was the same as in Experiment 1, except for the instructions regarding the charity. Before the experiment started, participants were told that they could earn extra money for themselves and for a charitable fund that they could choose themselves at the end of the experiment. After the experiment, they learned the details of three available charities to which they could select to donate their money. Apart from the Against Malaria Foundation, participants could also select the Give Directly Foundation or the Global Alliance for Improved Nutrition (GAIN): Salt Iodization Program to donate the money. All these three charities welcome small (i.e. €2) donations (see electronic supplementary material for complete information about the charity provided to participants). Participants could donate any amount of their earned money by putting coins inside an envelope labelled with the charity's name. The experimenter then left the cubicle. After the donation, the experimenter entered the cubicle and asked participants how they would spend the money earned for themselves to explore the idiosyncratic representation of the self-interest outcomes.^3^ The experimenter noted their answer. Finally, participants were debriefed. In total, participants donated €145.5 to the charities, which accounted for 121.3% of the initially reserved amount for donation according to the earnings in the learning phases (i.e. 60 participants * €2 for charity = €120).

#### 3.1.4. Data preparation and analyses

Like in Experiment 1, we trimmed the RT data of the correct responses in the test phase [42] by removing the RTs that were slower or faster than 3 s.d. of the participant's mean from analyses (4.0% of the RT data). We also did the reciprocal transformation to the remaining RTs and the accuracies, and we followed the same data analysis strategies as in Experiment 1. We predicted cue-based facilitation effects for both self-interest and other-interest outcome conditions, and the direction of the two effects should be opposite, i.e. a negative RT difference score in the self-interest outcome cue condition and a positive RT difference score in the other-interest outcome cue condition. Therefore, the coding for the contrast of RT difference was defined as follows: 0 for the RT difference (self minus other) in the neutral cue condition, −1 for the RT difference (self minus other) in the self-interest outcome cue condition, +1 for the RT difference (self minus other) in the other-interest outcome cue condition. The RT differences were subjected to a repeated ANOVA testing the contrast for the neutral, self-interest, and other-interest cues as a within-subject factor. For the analysis of accuracy difference, we reversed the contrast coding weight because participants should respond more accurately when the cue shares the identical outcome representation with the response.

### 3.2. Results

#### 3.2.1. Instrumental learning phase

The results indicate that no difference was found in RTs (*t*_57_ = 0.21, *p* = 0.835) and accuracy (*t*_57_ = −0.76, *p* = 0.449) in the instrumental training phase.

#### 3.2.2. Reaction times in the test phase

**Figure 4.**
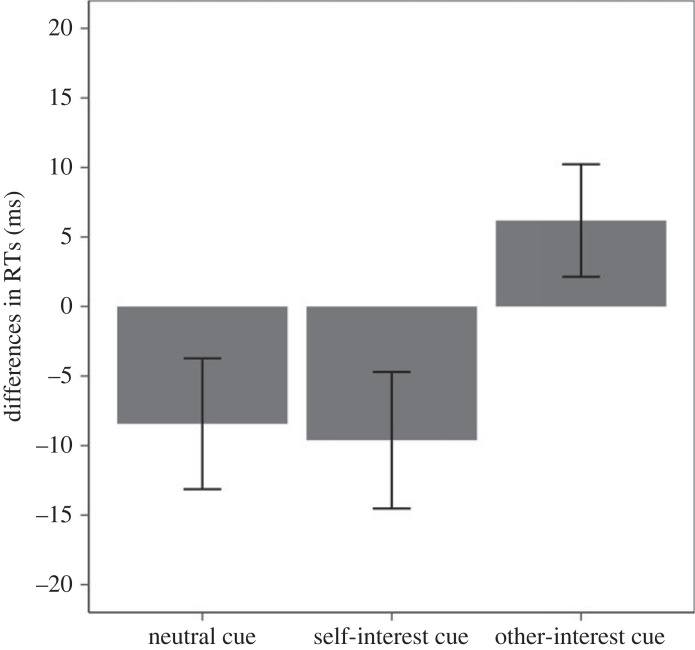
Experiment 2 RT difference in the three cue conditions (error bars represent one standard error). Note: a negative score represents faster self-interest responses, and a positive score represents faster other-interest responses.

The pattern of RT difference is presented in figure 4. The planned contrast was significant (*F*_1,57_ = 5.91, *p* = 0.018, $\eta_{p}^{2}=0.09$ [0.009; 0.228]). Although the neutral cue seemed to facilitate the self-interest response to some extent, the RT differences were in line with the predicted pattern: The self-interest outcome cue and other-interest outcome cue caused participants to respond faster, to respond with the corresponding outcome response, and their directions are opposite.

#### 3.2.3. Accuracy in the test phase

**Figure 5.**
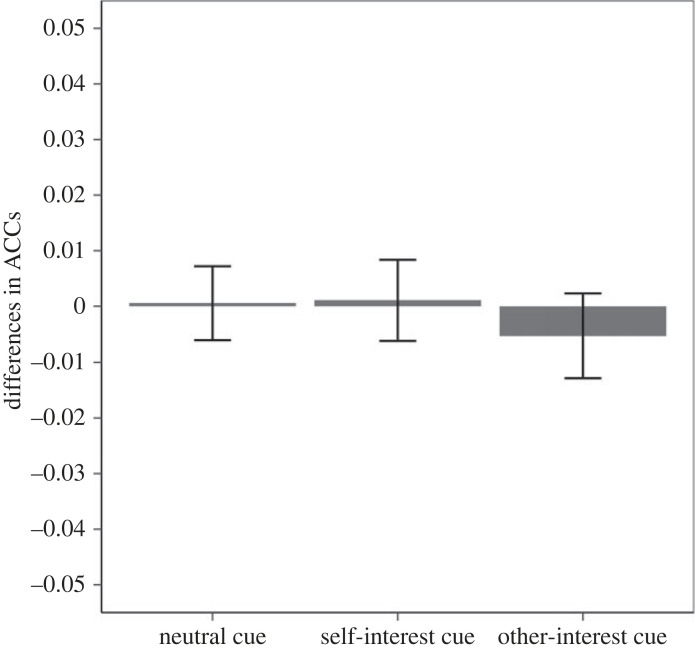
Experiment 2 accuracy difference in the three cue conditions (error bars represent one standard error). Note: A positive score represents more accurate self-interest responses, and a negative score indicates more accurate other-interest responses.

The pattern of accuracy difference is presented in figure 5. The planned contrast was not significant (*F*_1,57_ = 0.408, *p* = 0.526).

### 3.3. Discussion

In Experiment 2, we found evidence for a cue-based facilitation effect for self-interest outcomes as well as for other-interest outcomes. These findings differ considerably from those in Experiment 1, in which this effect only seemed to be present for the self-interest outcomes. Compared with the findings of Experiment 1, the other-interest outcome cue reversed the speed of executing other-interest responses. A clear difference between the two experiments concerns participants' ability to decide how to spend the money. Whereas Experiment 1 enabled participants to freely select an action for the self-interest outcome but not for the other-interest outcome, in Experiment 2, participants could freely choose how to spend the money in both outcomes. This pattern suggests that giving participants choice freedom over actions renders the other-interest outcome desirable as well in the task at hand. Circumstantial evidence for the relative importance of spending money on other-interest outcomes can be derived from the donation amount at the end of the experiment, showing that participants paid more to a self-chosen donation fund than they earned for themselves.

It should be noted that because we compared the cue-based facilitation effect of self-interest and other-interest outcome cues with a neutral cue condition, the results of Experiment 2 seem to show that the self-interest outcome cue only slightly increased the speed of self-interest actions compared with the neutral cue condition. One possibility could be that participants were generally faster in initiating the self-interest (compared with other-interest) responses, thus reducing the differences between the baseline and the self-interest cue condition. This observation resonates with the wealth of studies on the effects of self versus other representations in action fluency and action control [52–54]. Alternatively, participants might have explicitly compared the self-interest outcome with the other-interest outcome and concluded that the self-interest outcome is less valuable in the social context at hand. Importantly, whereas we do not know whether this process especially occurred in Experiment 2, the data still yielded the planned contrast effect on RT, revealing that self-interest and other-interest responses were both facilitated by the respective self-interest and other-interest outcome-related Pavlovian cues.

## 4. General discussion

The present study was set out to examine whether social goals and resulting actions can be controlled by environmental cues. People are highly motivated to seek rewards for self-interest purposes, while such motivation is commonly less strong when seeking rewards in the interest of others. By building on the PIT paradigm, we examined whether the subjective value of self- and other-interest outcomes can change the strength of the PIT effect. Employing a cue-based forced-choice response time task, the results of Experiment 1 indicated that cues associated with self-interest (versus other-interest) monetary outcomes caused participants to speed up instrumental action to attain the pro-self goal, even though participants had no direct sensorimotor experiences as to performing the action in response to the cues. This concurs with a goal-directed process that has been previously addressed in PIT studies [25].

Furthermore, while Experiment 1 showed a cue-based effect on pro-self goal pursuit but not on pro-social goal pursuit, we reasoned that such difference occurred because of the difference in freedom to spend the earned money: spending the money on oneself the way one likes versus spending the money only by donating to one charity (here the Against Malaria Foundation). As has been argued before, money is an all-purpose commodity that allows people to achieve several goals [55]. From this all-purpose perspective, our findings suggest that earning money for other-interest outcomes can be cue-based when people learn to have the freedom to use the monetary reward. Interestingly, research on consumer behaviour indicates that people prefer products that are considered to have more freedom (e.g. more functions) and judge them to be more worthy [56,57]. Considering the results from this view, the current study indicates that having a say in how to spend money on others renders pro-social goals more valuable, perhaps even more valuable in comparison with pro-self goals. This inference is further supported by the amount of money participants donated. In Experiment 2, they donated more coins than required, suggesting that they were more motivated to achieve the pro-social goal than the pro-self goal.

The present findings also speak to previous research on concept activation effects on behaviour, which relies on individual pre-existing knowledge about the action-relevant meaning of concepts [16,58,59]. This research argues when stimuli (such as words or pictures) are associated with a goal concept (compete, help) that is mentally represented as an outcome of action in a person's mind, these stimuli can trigger the goal and resulting action. Whereas this research may indicate that environmental cues can trigger action through activating social goals, this research does not rule out whether the stimulus evokes action directly, thus not excluding an S-R habit account. For instance, exposure to the word ‘competing’ or ‘helping’ might evoke motor activity available in a person's behavioural repertoire, but this does not necessarily mean that the action is driven by pro-self or pro-social goals directed at attaining self- or other-interest outcomes. Here we experimentally investigated and showed how the transfer from a Pavlovian cue to instrumental action occurs as part of their shared overlap with the self- or other-interest outcome attached to both. Thus, the route from cue to action is assumed to depend on the representation of the pro-self or pro-social goal that mentally lumps the cue and action together. The present findings, then, show that PIT provides an important additional test to examine whether cue-based behaviour is mediated by the representation of human goals, such as pro-self and pro-social goals that were examined here.

Although our findings suggest that the PIT forced-choice task is a promising tool to assess the occurrence of automatic social goal pursuit, a few important notes are in place. First, our approach differs substantially from the common approach in PIT research. In most PIT studies with human subjects, different instrumental responses (e.g. pressing a left or right button) and Pavlovian cues (e.g. a blue or red light) are linked to different outcomes (e.g. obtaining popcorn or crisps). Thus, outcomes contain not only the perceptual properties of the outcome (e.g. size, colour, taste) but also the motivation derived from the value of the outcome [18]. Value-based specific PIT effects are then tested by devaluing one of the outcomes (e.g. making popcorn taste unpleasant) or by comparing two outcomes of different values (popcorn versus tomato; monetary reward of 10 cents or 50 cents). In the present study, we used one single rewarding outcome (a 10 Euro cent coin) and reasoned that, in principle, attaining this reward has higher subjective value being framed as a self-interest (compared with other-interest) outcome. However, although the established PIT effect supports this idea, we did not devalue the outcome or measure the subjective value directly. This shortcoming is particularly an issue in Experiment 2, in which we expected that both the pro-self and pro-social goals would be triggered by the associated Pavlovian cues. The fact that only the pro-social goal showed PIT effects suggests that the self-interest outcome was devalued in the task at hand. Furthermore, the findings of Experiment 2 also show that the neutral cue (baseline) condition resembles the effect of the self-interest cue condition, suggesting that the absence of the pro-self goal effect could be due to changes in the baseline condition (see discussion of Experiment 2).

Furthermore, the typical PIT methodology for studying human behaviour is to test the effects of cues in free-choice settings that target decision-making processes. Although Pavlovian cues have the potential to evoke goal-directed decision-making, earlier, we argued that the decision responses are open to disturbances from free choice and task-strategic processing. We aimed to circumvent this issue by designing a PIT test that employs a forced-choice speeded task (see also [38,39]). Forced-choice tasks provide the opportunity to test the influence of cues by creating response facilitation situations, as is typically done in response priming [60] or Simon tasks (e.g. [61]). The logic is simple: when a cue triggers a response that one is instructed to perform, a response speed-up arises. Thus, integrating PIT research with forced-choice speeded tasks allows us to test how specific responses that are instrumental in attaining specific outcomes (low- versus high-value outcome) are evoked by the Pavlovian cues associated with these outcomes. Importantly, a limitation of such a task is that one can only look at the initiation of actions and not the motivation to engage in them. Hence, a test methodology that relies on the speed of responding could be further developed, including a measure of motivational strength, such as investing effort in action performance once action initiation takes place. In fact, original studies on PIT with animals considered (albeit implicitly or explicitly) the degree of motivational strength as an essential part of the PIT test, as being operationalized by action intensity and persistency in the presence of Pavlovian cues [62,63].

Finally, the current findings indicate that cue-based goal-directed behaviour is sensitive to the social meaning of rewarding outcomes at stake. In so doing, our findings may stimulate PIT research to take a closer look at the role of social cognition. For example, an essential root of other-interest behaviour is the human capacity to empathize and understand other people's mind [64–67]. People care about others and are empathic in predicting and emotionally evaluating the consequences of their actions for others. The PIT paradigm may be helpful to further our understanding of the human nature of social behaviour and to generate testable hypotheses relevant to how other-interest outcomes are learned, represented and expressed in the presence of environmental cues. An intriguing and important direction for future research might be testing whether and how the effects of choice freedom and empathy interact in shaping other-interest behaviour as a result of PIT, because, under such conditions, people might be able to freely put their own concerns aside to help others in the way they like.

## Data Availability

The dataset and analyses code are available through the Open Science Framework website at the following link: https://osf.io/cb5kz/?view_only=eb87fe5d61e542d2a73c19188988b123. The data are provided in electronic supplementary material [68].

## Footnotes

1 Although we used transformed data to feed the analysis, for clarifying the predicted pattern, figures of the RTs and the accuracies in both experiments were presented with untransformed data. The omnibus *F* test and the following *post hoc* test are reported in the electronic supplementary material.

2 The DVs used in all analyses have been reciprocally transformed.

3 The records of participants' self-interest outcome can be found at https://osf.io/cb5kz/?view_only=eb87fe5d61e542d2a73c19188988b123, and the donated coins have been donated to relevant charities after completing all data collection.
